# Cyanide and Cyanogenic Compounds—Toxicity, Molecular Targets, and Therapeutic Agents

**DOI:** 10.3390/biom14111420

**Published:** 2024-11-07

**Authors:** Joanna Izabela Lachowicz, Jan Alexander, Jan O. Aaseth

**Affiliations:** 1 Department of Population Health, Division of Environmental Health, Occupational Medicine and Epidemiology, Wroclaw Medical University, Mikulicza-Radeckiego 7, PL 50-368 Wroclaw, Poland; joanna.lachowicz@umw.edu.pl; 2 Norwegian Institute of Public Health (NIPH), N-0213 Oslo, Norway; jan.alexander@fhi.no; 3 Department of Research, Innlandet Hospital Trust, N-2381 Brumunddal, Norway; 4 Faculty of Health and Social Sciences, Inland Norway University of Applied Sciences, N-2418 Elverum, Norway

**Keywords:** cyanide, cyanogenic compounds, poisoning, mitochondria, iron(III), methaemoglobin, cobalamin

## Abstract

Cyanide (CN) is a well-known mitochondrial poison. CN poisoning may result from acute or long-term exposure to a number of CN compounds. Recent insight into the chemical affinities of the CN anion has increased our understanding of its toxicity and the mechanisms of antidotal actions, which, together with information on various exposure sources, are reviewed in the present article. A literature search in Scopus, Embase, Web of Science, PubMed, and Google Scholar for the period 2001–2024 revealed that the CN anion after exposure or degradation of CN compounds is distributed to vulnerable copper and iron-containing targets, especially in mitochondria, thus blocking the electron transport chain. Intake of cyanogenic compounds may exert subacute or chronic toxic effects, also because of the interaction with cobalt in vitamin B_12_. Antidotal agents exert their effects through the affinity of CN for cobalt- or iron-containing compounds. Research on CN interactions with metalloproteins may increase our insight into CN toxicity and efficient antidotal regimens.

## 1. Introduction

Cyanide (CN) exposure may kill organisms from insects and fish to mammals, including humans, within seconds to hours [1]. Cyanide poisoning may result from acute or long-term exposure to several cyanide forms, including the gas, HCN (e.g., fumes of fires), liquid (e.g., contaminated water), or solid (e.g., plant food products with cyanogenic glycosides from which cyanide can be released) state. CN has also been widely used for criminal acts [2].

CN is a mitochondrial poison, and cyanide ions interfere with iron enzymes in the mitochondrial respiratory chain, resulting in the block of aerobic respiration. Thus, early symptoms of acute toxicity include headache, tachycardia, and dyspnea [3]. Neurological symptoms with seizures, as well as bradycardia and falling blood pressure, usually accompany acute poisoning. Loss of consciousness followed by cardiac arrest may occur within minutes [4]. The toxicity onsets and progression depend on cyanide form and dose, time of exposure, route of intoxication, and health conditions of the individual. An individual who has survived acute cyanide poisoning may still suffer from neurological and/or psychological dysfunction for months or years.

The diagnosis of cyanide toxicity is difficult, as the multitude of symptoms are unspecific. The acute cyanide intoxication can be diagnosed upon analysis of the incident’s place. Blood analyses of cyanide are difficult, while free cyanide and its immediate metabolites in blood are highly unstable. Chronic exposure to cyanide gives mild symptoms at the beginning of exposure, which may increase with time. The diagnosis is complicated since there are no specific biomarkers for chronic cyanide toxicity.

The aim of the present article is to give an update of cyanide exposure sources, molecular affinities and targets of cyanide anion, symptoms of acute and chronic poisoning, and recent advances as regards diagnosis and antidotes against its toxicity. The literature search was made in Embase, PubMed, Scopus, Web of Science, and Google Scholar databases, with the time-range filter from 2001 to 2024. The literature selection was prepared separately for each paragraph with the use of proper search terms.

## 2. Cyanide Exposure Sources

**Figure 1:**
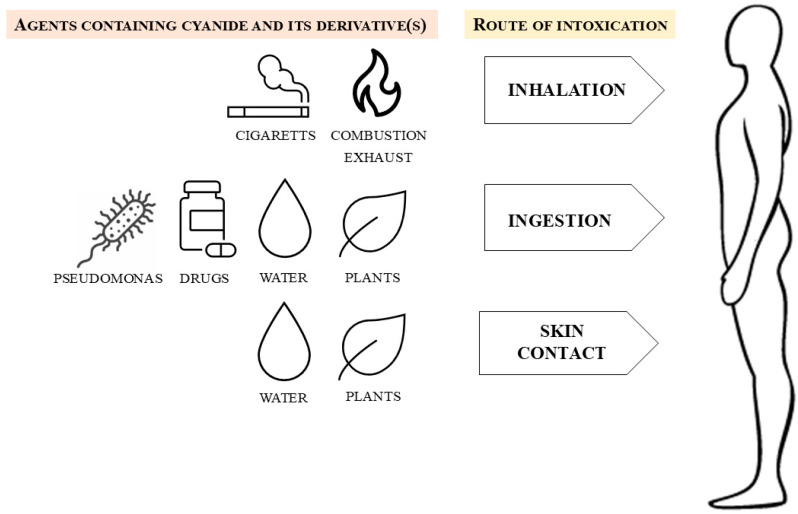
Routes of cyanide (CN) exposure or its precursors/derivatives may occur via inhalation and skin contact with combustion smoke (fabrics or biological samples containing nitrogen and carbon atoms) or via ingestion (accidental or during murder/suicide attempts) of contaminated water and cyanogenic food. The acute lethal oral dose of cyanide in humans is reported to be between 0.5 and 3.5 mg/kg bodyweight (BW). The toxic threshold value for cyanide in blood is considered to be between 0.5 mg/L (ca.20 µM) and 1.0 mg/L (ca. 40 µM); the lethal threshold value ranges between 2.5 mg/L (ca. 100 µM) and 3.0 mg/L (ca. 120 µM).

Toxic cyanide is present in the form of hydrogen cyanide (HCN) gas, different cyanide salts, and organic compounds. There are numerous sources of environmental cyanide products (Figure 1), among which smoke from house fires of nitrogen and carbon-containing polymer materials is a relatively common cause of acute intoxication [5]. In addition, some insecticides are potential cyanide sources [6]. Nitroprusside used as a drug for hypertensive crisis can break down into nitric oxide and cyanide, which may lead to toxic effects [6]. Cyanide from tobacco alkaloids is present in tobacco smoke, thus leading to a modest chronic exposure in the smoke [7]. Moreover, cyanide can be a metabolite product of some bacteria strains living in the environment or human body [8].

Cyanide exposure with increased uptake in blood and toxicity can occur from open fire during various conditions, while toxicity from an enclosed fire is often additive to CO intoxication [9]. The blood CN concentration exceeding 20 µmol/L (0.5 mg/L) is believed to be toxic, while concentrations ranging from about 100 to 120 µmol/L (2.5–3 mg/L) and above may be lethal [10,11,12].

Volatile cyanide compounds are combustion products of meat frying and grilling in households and restaurants and constitute a chronic cyanide exposition, which is underestimated. Moreover, surgical smoke contains volatile cyanide compounds [13], leading to the chronic exposition of operating room personnel.

The exposure from cyanogenic foods is generally higher than exposure from inhalation or drinking water [14]. Data on acute lethal toxicity of cyanide (LD_50_) and different inorganic salts obtained in rat studies [15,16,17,18] vary from 4 mg/kg for HCN to 1265 mg/kg for CuCN. In humans, lethal doses range from 0.5 to 3.5 mg/kg of body weight (BW) [19]. Median oral LD_50_ for various cyanogenic glycosides (linamarin, prunasin, and amygdalin) in experimental animals ranges from 450 to 880 mg/kg.

There are different cyanide health-based guidance values established by competent agencies [14,19,20]. The European Food Safety Authority (EFSA) in 2016 established (and in 2019 confirmed) an acute reference dose (ArfD) for CN of 20 μg/kg BW/day [10]. Notably, EFSA stated that data from human and animal studies were not sufficient to establish a chronic health-based guidance value (HBGV) for cyanide [12].

**Figure 2:**
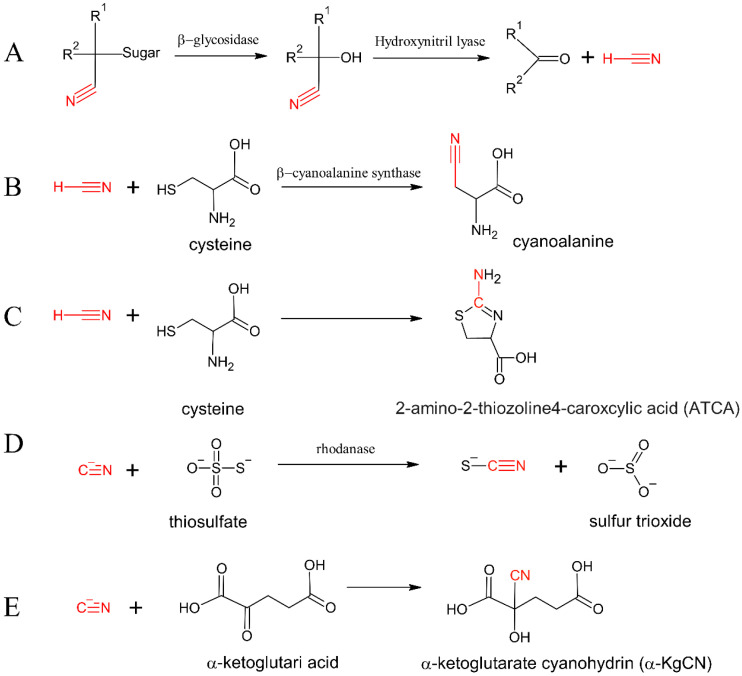
Release of cyanide from cyanogenic glycosides (CNGs) and biotransformation of cyanide. R^1^ = phenyl/p-hydroxyphenyl/methyl and R^2^ = hydrogen/methyl/ethyl, substituents, sugar = glucose (S or R)/gentobiose (R). Release of cyanide: (**A**); detoxification: (**B**), cyanoalanine, (**C**) 2-amino-2-thiozoline4-caroxcylic acid (ATCA), (**D**), thiocyanate and (**E**) α-ketoglutarate cyanohydrin (α-KgCN).

Plant-derived food containing so-called cyanogenic glycosides (CNGs; Figure 2A) is a major source of cyanide. The general structure of CNGs contains a central carbon attached to a -CN moiety and two groups (R1: methyl, phenyl, or p-hydroxyphenyl group; R2: hydrogen, methyl, or ethyl group) and a monosaccharide (glucose) or a disaccharide (gentiobiose) unit.

There are several thousand plant species that contain cyanogenic compounds; among these food plants are cassava root, sorghum leaves, linseed meal, lima beans, bamboo shoots, apple seed, stone fruit kernel, peach apricot, plum, nectarine, (bitter) almond, and cherry. CNGs are secondary plant metabolites thought to be part of a defense mechanism [20]. They are found as an α-hydroxynitrile bound to a β-glycoside of a mono- or disaccharide such as, e.g., glucose or gentiobiose [20]. Upon destruction of the plant cell (following maceration or wounding of the plant, or by gut microflora), CNGs are brought in contact with glycosidases, which hydrolyze the glycoside bond followed by release of cyanide. The glycoside is enzymatically converted to the corresponding cyanohydrin, which then spontaneously decomposes to form HCN, ketone, or aldehyde (Figure 2A). Because of variable molecular masses, CNGs yield different amounts of cyanide per gram.

Cyanide can be detoxified through several metabolic pathways, including those outlined in Figure 2B–D. The main pathways of CN detoxification are conversion to thiocyanate in the presence of a sulfur donor, for instance, cysteine amino acids (Figure 2B,C), thiosulfate (Figure 2D), or α-ketoglutaric acid (Figure 2E).

Cassava (*Manihot esculenta*) is a staple food for over 800 million people in Africa, Asia, and America [21]. It contains different CNGs (principally linamarin and lotaustralin), which upon hydrolysis release HCN or cyanide anion. Cassava poisoning may result in death or irreversible neurological deficits, such as the paralytic disease known as konzo (*kiyaka* in the local language, which means “tied legs” [21]. Traditional or industrial processing methods eliminate cyanogenic glycosides and their metabolites before cassava consumption. However, during climate or social disaster periods (e.g., flood or war), such elaborations may be omitted by poor populations [21].

The experiments conducted with rat models showed that animals receiving linamarin (oral dose of 250 or 500 mg/kg BW) died within a few hours. Of note, the mortality rate in the group of animals supplemented with methionine was significantly lower. Thus, suggesting the cyanide detoxification role of methionine following the formation of low-toxicity compounds [22]. Another antidote for cyanide toxicity is sodium thiosulphate, which undergoes an enzymatic reaction with rhodanase in mitochondria and forms a less-toxic compound (Figure 2D) easily eliminated via urine [23]. The exhaustive review on metabolism and toxicity of cyanogenic glycosides in humans and animal models has been published recently by Cressey and Reeve [24].

Cyanides can be found occasionally at low concentrations in drinking water. However, there are industry incidents leading to the release of spills of cyanide into water. Moreover, cyanogen chloride can be formed as a by-product of chloramination or chlorination of water. It is slowly hydrolyzed by water at neutral pH to release cyanate and chloride ions, while in the human body it is metabolized to cyanide anion.

Cyanide baths and cradles are used to liquify silver and gold from the ore during the silver and gold mining activity. When practiced in the vicinity of agricultural areas, CN can be released into the environment [25,26], thus contaminating water and soil.

Considering a significant number of cyanide environmental and industrial sources, there is an urgent need for fast, economic, and efficient remedy methods. Recently, Kuliahsari et al. [27] and Alvillo-Rivera et al. [28] presented exhausting literature reviews on biological treatments with the use of enzymatic reactions for the degradation of cyanide.

Isocyanic acid (ICA), methyl isocyanate (MIC), and HCN are generated when protein and amino acids of meat and egg are heated during food preparation by cooking or frying [29]. Monoisocyanates (i.e., ICA and MIC) are electrophilic compounds of high reactivity, which may cause post-translational modifications in the body and lead to the proteins’ damage and dysfunction [30]. Kitchen staff can be exposed to cyanide volatile compounds of varying concentrations, from insignificant to toxic, depending on cooking type, type of food, and heating system, as well as materials used for cooking and the conditions in the kitchen itself [30]. Similar hazards, even if on a lower scale, could be present in surgical rooms, where surgical smoke is generated from electrocautery instruments [31].

Widespread access to internet shopping facilitates the purchase of highly toxic substances, including cyanides. For example, there have been several incidents of poisoning from cyanogenic glycosides in apricot kernels that have been marketed, i.e., as a remedy against cancer [12]. In the last two decades, the American Association of Poison Control Centers reported a 10% increase in cyanide-related suicide attempts by intentional cyanide ingestion [32], i.e., organic and inorganic cyanides such as acetone cyanohydrin (2-hydroxyisobutyronitrile). Postmortem analysis showed elevated concentrations of cyanide in the blood and gastric contents of victims. The analysis of whole-body distributions of cyanide showed the highest content in the lung, the heart, and the liver. The way of administration, chemical composition of the cyanide substance, time of exposure, route of intoxication, and quantity taken influence the toxicokinetics and toxicity of cyanide in the human body [33].

## 3. Molecular Targets of Cyanide Anion

**Figure 3:**
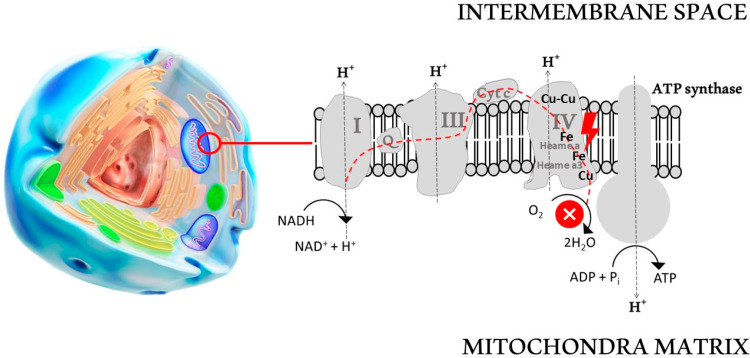
Regarding the consecutive complexes I–IV in the electron transport chain, cyanide poisoning results in an arrest in the electron flow to complex IV (which depends upon heme as a cofactor). CN^−^ has a high affinity for the ferric iron (heme a3) on cytochrome c oxidase, forming an adduct, which formation leads to inhibition of the electron transport chain.

The toxicity of cyanide results from its chemical bindings to iron and copper in cytochrome c oxidase in mitochondria [29]. This leads to a blockage of the electron transport chain and consequently an inability to produce ATP [34]. Cytochrome c oxidase is the terminal enzyme in the respiratory electron transport chain, being responsible for the reduction of O_2_ to two H_2_O molecules. In the electron transport chain, cytochrome c oxidase receives electrons from complex III using cytochrome c as an electron carrier [35] (Figure 3).

Cytochrome c oxidase represents the essential part of complex IV, which contains two heme groups, a and a3, each coupled to redox-active copper ions, CuA(2Cu) and CuB(1Cu), respectively. Complex IV has an O_2_ molecule tightly bound between iron a3 and CuB atoms until four electrons are transferred to one O_2_ molecule, converting it into 2 H_2_O. A heme unit with an iron atom commuting between the ferrous and the ferric states in cytochrome c operates as electron transporters from complex III to complex IV. In complex IV, CuA is a first electron acceptor, which transfers electrons successively to heme a that further passes electrons onto heme a3/CuB [36].

The balance of the reaction is that 4 protons reduce O_2_ to two water molecules, and Fe(II) is oxidized to Fe(III). As regards the cyanide interaction with the heme moieties, the CN^−^ anion blocks the enzymatic electron transfer through heme binding to Fe(III) in heme a3 (Figure 3).

CN^−^ binds to heme a3 both in the oxidized and reduced form, but more firmly to the oxidized form. However, binding appears to occur more rapidly to the one-electron reduced heme a3/CuB complex during turnover of the cytochrome c oxidase. Physiological concentrations of cyanide stimulate mitochondrial complex IV and enhance cellular bioenergetics [37].

As regards the affinity of CN^−^ to Fe(III) and Cu(II), CN^−^ can be considered an intermediate electron donor, whereas Fe(III) and Cu(II) are intermediate electron acceptors according to the Hard and Soft Acids and Bases (HSAB) theory of Pearson [38,39] (Table 1). The particular binding properties of CN also follow from their electronegativity values. Nitrogen in cyanide with a value of about 3 has a high tendency to donate electrons, whereas Fe(III) with a value of about 1.8 represents the electron acceptor in the interaction.

Notably, in addition to cyanide, H_2_S, CO, and NO inhibit cytochrome c oxidase by binding to the heme a3/CuB complex, while NO may effectively displace CN [37,40,41,42]. But the interaction is concentration dependent, as at low levels NO may even enhance cyanide inhibition [34].

**Table 1:** Classification of selected electron donor groups (bases) and electron accepting metal ions (acids) according to the hard and soft acids and bases (HSAB) theory of Pearson (Aaseth et al., 2016; Pearson, 1995 [43,44]).

Electron Donor Groups (Bases)			Electron Accepting Metal Ions (Acids)		
Hard	Intermediate	Soft	Hard	Intermediate	Soft
H_2_O, OH^−^, CO_3_^2−^, NH_3_, PO_4_^3−^	RNH_2_, **CN**^**−**^	RSH, RS^−^, **SCN**^**−**^	Na^+^, K^+^, Ca^2+^, Mg^2+^, VO^2+^, Mn^3+^, Al^3+^, Ga^3+^, Cr^3+^	Zn^2+^, Pt^2+^, Cu^2+^, Fe^3+^, Co^+^, Fe^2+^, Pb^2+^, Bi^3+^, Cr^2+^, Mn^2+^, V^2+^, Ni^2+^	Hg^2+^, CH_3_Hg^−^, Cd^2+^, Ag^+^

As can be deduced from Table 1, cyanide forms complexes with several metals such as platinum, zinc, copper, cobalt, iron, and nickel. The stability constants of some common metal cyanide species are presented in Table 2. The stability constants are listed in terms of log K, where K is the constant for the equilibrium of the metal–cyanide anion complex formation. The stability of the cyanide complex is growing with the higher log K value. Thus, the iron cyanide complexes are the most stable. Mercury, copper, nickel, and silver form medium-strength complexes, while cadmium and zinc form weak complexes.

## 4. Toxicokinetics and Factors Affecting Cyanide Toxicity

The cellular mechanism of cyanide toxicity depends on the route of intoxication. Thus, the clinical outcomes based on the toxicokinetics and toxicodynamics of oral cyanide are distinct from those of inhaled cyanide. As pointed out by Hendry-Hofer et al. [43], oral CN intoxication may lead to higher absorption than following CN inhalation. Patients exposed to oral CN can absorb toxins continuously. On the contrary, inhaled exposure relies on the individual’s respiration rate and is limited to the development of apnea from cyanide toxicity.

Analysis of the toxicokinetic behavior of cyanide and its metabolism products delivers insight into the suitable biomarkers of cyanide exposure [44] and targets for cyanide intoxication therapy.

The available toxicokinetic data are obtained on different animal models. However, the metabolism of cyanide and its main metabolite, thiocyanate, is species-linked, with some animals (e.g., goat) being more vulnerable to the toxic effects of cyanide and thiocyanate [45] (Table 3). Of note, age and gender of the animal influence significantly the toxicokinetic results. However, these data are often omitted in experimental data descriptions.

The studies conducted with a mouse model (single oral dose of KCN in water) were used to investigate the dose–response correlation and toxicokinetic characteristics [47]. Toxicokinetic analysis showed rapid CN uptake, metabolism, and clearance of plasma cyanide. Elimination CN half-time (hours) in adult female mice was faster than in males. However, the gender difference in elimination half-life time was not observed in juvenile mice.

Dirikolu et al. [50] presented results of toxicokinetics of cyanide in horses. The toxicokinetics were separated into two distinct phases. In the first phase (acute), CN was metabolized fast into SCN. In the second phase (chronic), the CN toxicokinetics were dominated by large CN distribution volume and long plasma half-life (12–16 h).

In the study by de Sousa et al. [46], the toxicokinetic parameters of SCN after oral administration of potassium cyanide (KCN) to female rats at diestrus, gestational, and lactational periods were investigated. The presented data showed that SCN concentrations were higher in serum, milk, and amniotic fluid after administration of potassium cyanide. The CN metabolism varied between female rats at different physiological states.

Mitchell et al. [44] analyzed the toxicokinetics of α-KgCN (Figure 2E) and compared this metabolite behavior with other cyanide metabolites in the plasma of 31 Yorkshire pigs. The maximum concentrations of α-KgCN and cyanide were relatively low (2.35 and 30.18 µM, respectively), thus suggesting that only a part of CN is metabolized to α-KgCN. However, the α-KgCN concentration increased more than 100-fold over compared to endogenous concentrations. At the same time, ATCA increased only three-fold. The results of this study showed that the use of α-KgCN as a biomarker for cyanide exposure could be a new strategy in analytical techniques of cyanide quantification.

The studies on rat models by Djerad et al. [51] showed that respiratory acidosis favors the cerebral distribution of cyanide. Moreover, respiratory acidification and alkalinization influence significantly the distribution of intravenously administered cyanide into the brain. In a control model with normal respiratory, the CN distribution is extremely fast (whole blood T_1/2_α = 21.6 ± 3.3 s) with an apparent volume of distribution of 0.83 l/kg.

Upon exposure, the CN anion is easily absorbed from the respiratory tract, and it is also taken up from the gastrointestinal tract. CNGs food may release CN already upon food processing or in the gastrointestinal tract, depending upon enzymatic activity in the gut wall or in the microbiota.

The kinetics of decomposition of plant cyanogenic glycosides to hydrogen cyanide in the human gut were reviewed recently by Cressey and Reeve [24]. Of note, a large number of reviewed studies were quite old, with the use of non-specific analytical techniques. As pointed out by the authors, new studies with the use of modern chromatographic methods to identify metabolites of the cyanogenic glycoside are needed.

As for the fate of ingested intact cyanogenic glycosides, few studies are available. Apparently, the relative amounts excreted as intact cyanogenic glycosides or as cyanide metabolites vary. For example, when pure linamarin, the main glycoside in cassava, was given to rats, 20% was excreted unchanged and 12% as a cyanide metabolite [22].

Once in the bloodstream, HCN (pK_a_ = 9.21) reacts reversibly with available electron acceptors, including the minute amounts of methemoglobin, and free cyanide is transported rapidly with erythrocytes to all tissues. Cyanide metabolism occurs mostly in the liver (80%), where the sulfurtransferase enzyme is present in the mitochondria. The formed thiocyanate ions are considerably less toxic than cyanide. Nearly 15% of cyanides react with cysteine to form ATCA [13] (Figure 2C). Other pathways of cyanide metabolism occur in different modes, among which its binding to vitamin B_12_ (leading to the formation of cyanocobalamin) is of particular interest. The remaining cyanides are oxidized to formate (excreted into urine) and carbon dioxide (excreted by the lungs). Already a few hours after the cyanide exposure, no free cyanide in the blood can be detected due to the rapid metabolism.

It is well known that nutritional deficiency may aggravate toxicity of CN from food containing CNGs, as has been observed upon long-term intake of cassava. Inadequate supply of sulphur amino acids would compromise CN detoxification, and a diet low in vitamin B_12_ reduces the formation of less toxic cyanide complexes. Epidemiological studies have evidenced that women and children consuming CNG containing food combined with low intake of food items of animal origin, and thus a marginal or deficient status of cobalamin and sulphur amino acids, are most often affected by the disease konzo [52].

Thiocyanate is a potential thyroid disruptor due to its capacity to block the uptake of iodide by the thyroid gland. Quantification of the dose–response relationships of SCN and changes in thyroid hormone concentrations are of paramount importance in evaluating the risk of exposure to SCN in humans and was recently reviewed by Willemin et al. [53]. The interactions between the numerous transporter- and enzyme-mediated modes of action increase the complexity of the dose–response relationship determinations for SCN.

## 5. Symptoms of Acute and Chronic Poisoning

### 5.1. Acute Exposure

The clinical outcomes of inhaled cyanide toxicity result within seconds after exposure, whereas symptoms of cyanide ingestion occur in minutes to hours [20]. High-concentration inhaled CN can lead to dyspnea, respiratory depression, apnea, hypotension, arrhythmias, coma, and seizures (Table 4). Increased venous hemoglobin oxygen saturation often appears as a pink skin tone. Low-level cyanide inhalation leads to weakness, vertigo, and confusion, which can be followed by loss of consciousness, dizziness, headaches, and difficulty breathing.

Initial symptoms of cyanide poisoning are non-specific and include anxiety, confusion, headache, and abdominal pain [54]. Compared to inhaled cyanide exposure, where dyspnea appears a short time after exposure, the onset of outcomes of oral cyanide exposure is not immediate [55]. Patients with cyanide ingestion often present tachypnoea, tachycardia, hypertension, and lactic acidosis, which rapidly progress to respiratory and circulatory collapse.

Ingested inorganic salts of potassium cyanide (KCN) or sodium cyanide (NaCN) are rapidly hydrolyzed into HCN in the acidic pH of the stomach. This cyanide form easily passes the cell membrane and inhibits aerobic metabolism in mitochondria.

The clinical symptoms of acute and chronic cyanide toxicity are listed in Table 4.

### 5.2. Chronic Exposure

Chronic exposure to low cyanide levels (e.g., consumption of processed apricot kernels [56]) may be accompanied by modestly raised blood CN concentrations. Nevertheless, this might be associated with hypoxia [56], leading to headache, dizziness, mild confusion, abdominal cramping, nausea, and vomiting, and further toxic myelopathy with permanent paralysis (e.g., in the form of the konzo disease) and other nervous lesions such as tropical ataxic neuropathy. These conditions are mostly seen when cyanide exposure occurs in combination with nutritional deficiency, i.e., deficient intake of sulphur-containing amino acids, iodine, and/or vitamin B_12_. The mechanisms of these pathologies are not fully understood; they have been attributed to chronic exposure to cyanide released from CNGs but also to various low molecular weight nitriles in cassava and lathyrus [57].

Reports on (acute) toxicity and death due to consumption of cassava present the following symptoms: dizziness, headache, lethargy, and even seizing episodes. Among gastrointestinal effects are listed abdominal pain, nausea, and vomiting [58]. The subjects with severe Konzo present dysfunction in the motor system, with leg paralysis or even tetraparesis, where both legs and arms are paralyzed [21,59]. Symptoms at onset may occur during or after a prolonged walk and may lead to leg trembling, even in association with paresthesia, sensations of electrical discharges in the spine and legs, and loss of visual acuity. However, peripheral nerves are not involved. The thiocyanate metabolite, combined with iodine deficiency, may inhibit iodine transport and cause hypothyroidism. Among other effects seen following consumption of cassava are mild liver and kidney damage.

Exposure to cyanide in drinking water may give rise to hypothyroidism due to the inhibition of iodine uptake in the thyroid gland by thiocyanate generated through the detoxifying action of rhodanese. Recent studies showed that cyanide-induced endocrine defects are associated with downregulation of CREM gene expression and a decrease in some endocrine hormones (follicle-stimulating hormone, luteinizing hormone, and testosterone) [60].

Previous studies showed that cyanide may lead to hypothalamic/testicular and reproductive toxicities [61], but the toxic mechanism remains unknown. Cyanide-induced impairment of sperm function and infertility [62] was associated with cAMP-response-element modulator (CREM) signaling pathways during spermatogenesis.

Workers engaged in the large-scale processes of cyanide removal from cassava (i.e., flour production) could be potentially exposed chronically to HCN at levels exceeding the safety limits. Recent studies [63] showed that workers involved in cassava processing are under HCN chronic exposure ranging between 0.464 and 3.328 mg/m^3^ (time weighted average, TWA). This range is below the threshold limit value ceiling (TLV-C) (5 mg/m^3^). However, it is above the action level (2.5 mg/m^3^). In addition, the biological monitoring of the exposed population showed increased urinary SCN^−^ levels [63].

### 5.3. Diagnosis and Biomarkers

The CN toxicokinetics are fast, so quantitative methods to identify cyanide and its metabolites in biological fluids (blood, urine, breath exhale, saliva) need to be rapid. Route of intoxication and time after exposure influence the choice of proper biomarker and type of biological fluid to be analyzed.

Diagnosis of cyanide toxicity inhaled from enclosed fire is usually based on detection of high lactate (due to anaerobic ATP production) concentration in blood. Acidosis with lactate levels above 6 mmol/L, especially in the presence of alterations in consciousness, is indicative of cyanide poisoning [10]. Such acute poisoning often results from cyanide-containing fumes or smoke exposure from fires. Unfortunately, cyanide as a cause may easily be missed in such patients. Thus, there is a need for updated guidance on cyanide concentrations that would be of concern for public health following short-term exposure. Of note, the data on acute exposure to cyanide are unsuitable for use in deriving a health-based value for short-term exposure due to the significant uncertainty of the available data.

Recently, Jackson and Logue published an exhaustive review on different analytical sensing technologies, which could be used to quantify cyanide in a rapid and field-portable way [64]. Colorimetric method after microdiffusion with a Conway cell [52] is an inexpensive qualitative analysis. However, it is perturbated by acidic conditions, which lead to the conversion of thiocyanate into cyanite. Other colorimetric direct methods of cyanide detection include the Prussian blue test, the pyridine-barbiturate assay, and the taurine fluorescence-HPLC [65]. However, the colorimetric methods lack the cyanide selectivity and are thus prone to false-positive results. The headspace gas chromatographic (GC) techniques were recently adopted for cyanide analysis [66]. However, also GC requires sample preparation (volatilization) under acidic conditions [67]. New electrochemical detection methods with sensitive cyanide-specific electrodes have been under development [68,69].

The significant difference between the toxicokinetics of CN and SCN stays in the t_max_. The CN t_max_ is much lower than SCN t_max_. Moreover, the onset of CN toxicity is fast. A relatively short t_max_ and large increase over baseline concentrations make cyanide itself the best diagnostic marker.

In cases of chronic exposure to ingested cyanide, much of the published research used concentration analyses of SCN in urine (U-SCN) or plasma (P-SCN) [52]. For instance, in the konzo-affected populations, the levels of U-SCN reached 1720 μmol/L and P-SCN 426 μmol/L, while 10 ppm (10 μmol/L) is considered a safe limit.

A new biomarker suitable for the analysis of cyanide detoxification products after consumption of cyanide glucosides is α-KgCN. As shown by Mitchella et al. [44], the α-KgCN concentration increases >100-fold over endogenous concentrations compared to only a three-fold increase for cyanide and ATCA.

Considering the low concentrations of CN and its metabolites (SCN^−^, ATCA, and α-KgCN derivatives and protein-bound cyanide adducts), these markers would necessitate a highly sensitive analytical method to diagnose exposure.

The new frontier in the diagnosis of CN toxicity could be mRNA analysis. Recently, Kim et al. [70] found that the following mRNAs: ADCY5, ANGPTL4, CCNG2, CD9, COL1A2, DACT3, GGCX, GRB14, H1F0, HSPA1A, MAF, MAT2A, PPP1R10, and PPP4C, were downregulated by sodium cyanide exposure both in in vitro (cell culture studies) and in vivo (Sprague-Dawley rats’ model) studies.

The experimental physiologically based pharmacokinetic (PBPK) model presented by Stamyr et al. [71] proved that breath HCN levels after inhalation exposure (0.1–1 ppm) increase one to two orders of magnitude with respect to the background breath level of about 0.01 ppm in unexposed subjects. Thus, breath analysis could be used in the future as a rapid diagnostic method for cyanide poisoning.

## 6. Therapy

Successful treatment of cyanide poisoning depends on the type and source of toxic cyanide exposure, together with adequate timing of the therapeutic intervention [72].

The current recommendations for therapy of acute cyanide toxicity imply the use of antidotes, which lower the concentration of cyanide in the blood and tissues by binding free cyanide in a complex and/or increasing its excretion in a non-toxic form.

Current approved treatments for cyanide intoxication can be divided into three classes: methemoglobin generators and nitric oxide donors (sodium nitrite, amyl nitrite, and dimethyl aminophenol), sulfur donors (sodium thiosulfate and glutathione), and direct binding agents (hydroxocobalamin and dicobalt edetate).

There are few therapies for inhaled cyanide toxicity, but any of the FDA-approved pharmacological therapies (namely hydroxocobalamin, sodium nitrite, and sodium thiosulfate) is well suited for oral toxicity of cyanide. Of note, the drugs approved for cyanide toxicity must be administered intravenously, thus they cannot be used in mass casualty settings in a massive way and in a short time [73]. Another complication is that compounds like hydroxocobalamin are not water soluble (27.3 ng/mL).

An oral therapy, which inhibits and/or prevents cyanide absorption in the gastrointestinal tract, is urgently needed. Such oral therapy could also be used as an alternative treatment for any cyanide-intoxicated patients before they develop severe toxicity (are still conscious and can swallow) and those who are exposed to cyanide hazards at low concentration but for a prolonged time.

The synthesis of approved and experimental therapies for cyanide toxicity is presented in Table 5 and further described.

### 6.1. Methemoglobin Generation

Historically, the first-line treatment for cyanide toxicity was amyl nitrite or sodium nitrite, which induce methemoglobinemia.

Already in 1989, Dutch presented a case study of a 9-month-old infant admitted to hospital in comatose condition due to a cyanide poisoning suspect. Administration of 4-dimethyl-aminophenol and sodium thiosulphate, two known methemoglobin inducers, permitted full recovery of the infant. In vitro studies have suggested that the antidotal mechanism of nitrite, at least partly, can be explained by the formation of high mitochondrial concentrations of NO that antagonize CN-induced cytochrome c oxidase inhibition [74].

Amyl nitrite is a well-known vasodilator and is therefore employed to treat heart disease and angina. Moreover, isoamyl nitrite formulations (INFs) were proposed as an antidote for cyanide poisoning (US patent number: US20150105474A1 patent). It acts as an oxidant to induce the formation of methemoglobin, which in turn can attenuate cyanide as cyanomethemoglobin. The alkyl group is generally unreactive, and the chemical and biological properties are primarily due to the nitrite (ONO) group. Recently, the study of SIAN nasal spray in healthy adults was registered on the clinical trials platform (ClinicalTrials.gov ID NCT05194358; 47 enrolled participants). The study started on 21 December 2021 and has been completed on 13 October 2023. However, the results have not been published yet.

MetHb can detoxify CN by the direct binding to Fe(III) in the blood and the formation of cyanmethemoglobin [64]. Simultaneously, cyanide is released from its binding sites in cytochrome oxidase. When used as cyanide antidotes, nitrites convert hemoglobin into methemoglobin. However, the use of nitrites can be dangerous since methemoglobin does not carry oxygen, and severe methemoglobinemia needs to be cured with methylene blue for restoration of the natural Fe(II)-hemoglobin [75].

### 6.2. Dicobalt Ethylenediaminetetraacetic Acid (EDTA)

The mechanism of action for cobalt-containing scavengers is the direct binding of cyanide anion to cobalt. Cobalt ions, which share some chemical properties with iron ions, can also efficiently chelate cyanide [39]. Dicobalt edetate (dicobalt-EDTA; drug name in Europe: Kelocyanor) forms a stable cobalt–cyanide complex [76] and provides an antidote effect faster than methemoglobin formation. However, the clinical superiority of methemoglobin formation has not yet been demonstrated. This may in part be attributed to the fact that cobalt complexes are toxic, and therapy with Kelocyanor following the wrong diagnosis of cyanide toxicity can lead to fatal results.

The early case studies on dicobalt edetate therapy were published in 1978 by Negler et al. [77]. Three employees with different degrees of CN exposure were treated with cobalt EDTA. The authors concluded that, because of the degree of patient symptomatology associated with the use of cobalt EDTA, this therapy should be applied only for patients with the most severe degree of exposure to CN⁻. Thus, nowadays dicobalt-EDTA is reserved only for patients with the most severe exposure to cyanide. Often, glucose is administered with dicobalt-EDTA to protect against cobalt toxicity [76].

### 6.3. Hydroxocobalamin

Hydroxocobalamin binds strongly to only one cyanide anion through its central Co(III)-ion by forming a non-toxic metabolite—cyanocobalamin, thereby reversing the mitochondrial dysfunction (with rare exceptions [78,79]). Hydroxocobalamin has now been approved in the USA and is available also in Europe as Cyanokit antidote kits [10]. It is a form of vitamin B_12_ that is also found in food. The affinity of cobalamin toward the cyanide anion resides in its electrophilic cobalt atom (electronegativity 1.88) in the center, since cobalt with 6 coordination valences has one available valence to bind cyanide strongly, releasing it from its cytochrome c oxidase binding.

Recently, Dang et al. [80] described a case study of a 73-year-old female with metastatic pancreatic cancer who developed cyanide toxicity from taking amygdalin. The patient was treated with 5 g of intravenous hydroxocobalamin over 15 min. One hour after the administration of hydroxocobalamin, the patient’s blood pressure improved, while 2 h after treatment, the lactic acid level decreased. Her mental status returned to baseline 3 h after the treatment.

In contrast to hydroxocobalamin, cobinamide (a derivative of vitamin B_12_) can bind two cyanide equivalents. Previous studies with cobinamide showed improved efficacy in multiple animals over hydroxocobalamin [55].

### 6.4. Oxygen Therapy

The International Program on Chemical Safety, upon issuing a survey (IPCS/CEC Evaluation of Antidotes Series), recommends the listed antidotal agents against cyanide: oxygen, sodium thiosulfate, amyl nitrite, sodium nitrite, 4-dimethylaminophenol, hydroxocobalamin, and dicobalt edetate (“Kelocyanor”) [81,82].

In addition, qualified first aiders are recommended to administer oxygen therapy using a bag valve mask, provided they have been trained in its usage. This approach is also recommended in carbon monoxide poisoning, which can occur as a co-exposure to fumes from burning flat complexes.

In 2006, Lawson-Smith et al. conducted clinical trials (register number: NCT00399100 and NCT00280579) on 20 subjects from fire accidents receiving hyperbaric oxygen (HBO) treatment for carbon monoxide poisoning. However, CN concentrations in blood from patients admitted to hospital with CO intoxication and smoke inhalation exposure did not differ significantly from controls. Accordingly, they were not able to detect any changes in CN concentrations in blood after treatment with HBO [5].

### 6.5. Experimental Therapies

Medinox (chelator–iron complex of N-methyl-D-glucamine dithiocarbamate; MGD, and its derivatives, US5858402 A patent) and NOX-51 are promising antidotal agents, which have low toxicity, are fast acting, and do not rely on the generation of methemoglobin.

Sodium thiosulfate binding mitigates the conversion of cyanide to thiocyanate utilizing the enzymes rhodanese or 3-mercaptopyruvate sulfurtransferase enzymes [83]. Cyanide scavengers (namely sulfanogen, tetrathionate, and dimethyl trisulfid) have shown promising results in remedies for cyanide toxicity [84]. Of note, these agents offer efficacious intramuscular injection (IM) therapies in large animal models. For instance, recent studies with the use of a swine model (female Yorkshire swine (Sus scrofa) weighing 45–55 kg) showed that oral administration of sodium thiosulfate (without and in combination with glycine) improved survival, blood pressure, respiration, and blood lactate concentrations in the animal model of acute oral cyanide toxicity (8 mg/kg KSCN saline solution delivered as a one-time bolus via an orogastric tube) [85].

Dimethyl trisulfide (DMTS) is a naturally occurring substance of low toxicity. DMTS has shown antidotal efficacy in cyanide poisoning and is thought to act as both a sulphur donor and as a methemoglobin inducer. Rice et al. [86] showed that DMTS (25 mg/kg) is an efficacious cyanide antidote in the murine model, which significantly increases survival with minimal side effects.

The metal compound cisplatin is a well-known chemotherapeutic agent for different tumors [87]. The binding affinity of the cyanide anion for the positively charged metal platinum is known to create an extremely stable complex in vitro (logβ_4_ = 62.3 determined for [Pd(CN)_4_]^2−^ complex obtained at 25 °C in 0.1 M NaClO_4_ ionic strength) [88]. Platinum complexes based on naturally occurring metabolites of platinum have demonstrated promise as a new class of cyanide countermeasures.

Recent in vivo studies by Nath et al. [1] in zebrafish, mice, and rabbits showed that platinum-based compounds (a panel of 35 cisplatin analogs) are effective antidotes for cyanide poisoning. The mechanism of action is based on complex formation between platinum and cyanide. Of note, the rescue of cyanide with cisplatin occurs only with the DMSO-treated material. These observations implicate a significant role for a sulfur ligand in the efficacy of the platinum-based cyanide scavengers.

Recently, Behymer et al. [89] have hypothesized that thioethers enhance cyanide scavenging rates, leading to improved increased efficacy while also offering nephrotoxic protection that can be attenuated.

## 7. Conclusions

Exposure to cyanide or its precursors/derivatives occurs especially via inhalation and/or via ingestion. Acute exposure may occur from many sources, such as fires affecting, e.g., polyurethane polymers, ingesting of foods containing high amounts of cyanogenic glycosides, as well as occupational exposure in, e.g., metal workers. Major sources of chronic exposures to the general population are from cigarette smoking and from cyanogenic glycosides in plant-derived foods such as cassava, bamboo shoots, and stone fruit kernels. Chronic toxicity may be aggravated by nutritional deficiencies, i.e., due to reduced detoxification capacity. Wide availability and rapid harmful effects make cyanide persisting criminal and accidental hazards.

In the respiratory tract, as well as in the gastrointestinal tract, the cyanide anion is easily absorbed. It reacts rapidly with the Fe(III)-heme moiety in methemoglobin. Its toxicity is due to inhibition of cellular respiration through binding to ferric iron in cytochrome c oxidase of the mitochondrial respiratory chain and thus blocked ATP production, which may affect all cells. Onsets of cyanide toxicity symptoms, in both acute and chronic form, are characterized by unspecific signs, and diagnosis may be difficult. Different biomarkers of cyanide exposure may help in the diagnosis.

Several therapeutic approaches have been used, which include oxygen therapy, binding of free cyanide to Fe(III) in methemoglobin, or to Co(III)-containing agents. Experimental therapeutics include, i.e., increasing detoxication capacity by sulfur compounds and cyanide binding to cis-platinum. The knowledge of cyanide sources, molecular targets, toxicokinetics, and factors affecting cyanide toxicity is essential to reduce exposure, recognize potential victims, diagnose properly, and apply adequate therapy.

## Figures and Tables

**Table 2:** Stability constants of selected intermediate accepting metal ions–cyanide complexes.

Name	Formula	Dissociation Constant (Log K)
Ferricyanide	Fe(CN)_6_^3−^	52.0
Ferrocyanide	Fe(CN)_6_^4−^	47.0
Tetracyanocuprate(I)	Cu(CN)_4_^3−^	27.3
Tetracyanonickelate(II)	Ni(CN)_4_^2−^	22.0
Dicyanoargentate(I)	Ag(CN)_2_^−^	21.0
Tetracyanocadminate(II)	Cd(CN)_4_^2−^	16.9
Tetracyanozincate(II)	Zn(CN)_4_^2−^	16.9

**Table 3:** The toxicokinetic parameters of cyanide and thiocyanate in different animal models. (C_max_ peak plasma concentration, T_max_ time of peak concentration; t_1/2_α half-life of elimination, Kel constant of elimination, Cl clearance, V_d_ apparent volume of distribution; AUC area under the plasma concentration—time curve calculated from t = 0 to t = infinity by the trapezoidal method). Gender (M = male; F = female).

Parameter	Route of Administartion	Matrix	Gender	Analyte	CN Dose (mg/kg)	C_max_ (uM/L)	T_max_ (min)	t_1/2_α (h)	K_el_	Cl (mL/h/kg)	V_d_ (l/kg)	AUC (µM/h)	Reference
Goats	oral (by gavage)	blood	M	CN	3	93	15	1.28	0.54	13.2	0.41	234.6	[46]
Mouse	oral	plasma	M	SCN	6	348	4	9.34		19.5		4200	[47]
Mouse	oral	plasma	F	SCN	6	318	4	7.41		20.7		4373	[47]
Mouse	oral	plasma	M	SCN	8	415	4	8.13		23.4		5079	[47]
Mouse	oral	plasma	F	SCN	8	439	4	6.61		22.3		5595	[47]
Mouse	oral	plasma	M	SCN	11.5	530	4	12.2		21.8		6835	[47]
Mouse	oral	plasma	F	SCN	11.5	692	4	8.77		21.5		7747	[47]
Pigs	oral (by gavage)	blood	M	CN	3	57	30	0.54	1.28	22.0	0.29	140.7	[46]
Rabbit	intramuscular injection	Plasma	not reported	CN	2.5	15		2.95					[48]
Rats	oral (by gavage)	blood	M	CN	3	89	15	0.64	1.08	22.7	0.35	136.2	[46]
Rats	injected subcutaneously	blood	not reported	CN	2	52		20					[48]
Rats	injected subcutaneously	blood	not reported	CN	4	60		22.83					[48]
Rats	injected subcutaneously	blood	not reported	CN	6	69		25.16					[48]
Rats	injected subcutaneously	plasma	not reported	SCN	2	100		42.17					[48]
Rats	injected subcutaneously	plasma	not reported	SCN	4	168		47.67					[48]
Rats	injected subcutaneously	plasma	not reported	SCN	6	158		50.17					[48]
Rats	tap water	blood	not reported	CN	1	25		14.1					[49]
Swine	infused intravenously	plasma	not reported	CN	1.7	30		26.9					[48]
Horse	infused intravenously	blood	not reported	CN	60	446		0.74		182	0.39		[50]

**Table 4:** Summary of main cyanide poisoning symptoms as an effect of acute or chronic cyanide exposure.

	Acute Poisoning	Chronic Poisoning
Neurologic	Dizziness	
	Headache	
	Anxiety	
	Confusion	
	Weakness	
	Perspiration	
	Flushing	
	Bright red retinal veins and arteries	
	Seizures	*Konzo* (motor system dysfunction, tetraparesis, sudden leg tremblings, parasthesia)
	Stupor	
	Paralysis	*Tropical ataxic neuropathy* (several neurological symptoms affecting the mouth, eyesight, hearing or gait)
	Coma	
Respiratory	Tachypnea	
	Dyspnea	
	Respiratory depression	
	Absence of cyanosis	
	Hypoventilation	
	Apnea	
	Noncardiogenic pulmonary edema	
	Respiratory arrest	
Cardiovascular	Transient hypertension with reflex bradycardia and sinus dysrhythmia	
	Tachycardia	
	Hypotension	
	Elevated or depressed ST segment or a shortened ST segment with fusion of the T wave into the QRS complex	
	Atrioventricular block, erratic supraventricular rhythms, ventricular fibrillation, asystole	
	Cardiovascular collapse	
Other		Goitre and cretinism (due to inhibition of iodine transport by cyanate)
		Infertility

**Table 5:** List of current and experimental therapies for cyanide toxicity. * The CAK therapy is composed of 3 components: amyl nitrite, sodium nitrite, and sodium thiosulfate.

Name	Formula	Status	Administration
Sodium nitrite *	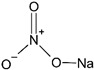	Approved	Slow IV injection at a rate of 2.5 to 5 mL/minute
Amyl nitrite *	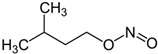	Approved	0.3 mL by inhalation of crushed ampule, may repeat 3–5 min
Sodium thiosulfate *	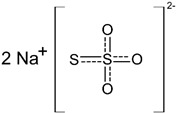	Approved	50 mL (12.5 g/100 mL) of sodium thiosulfate injection immediately following administration of sodium nitrite
Dicobalt edetate	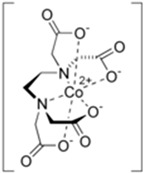	Approved	300 mg (1 ampoule) IV injection over 1 min, followed by 50 mL of 50% dextrose IV to protect against toxicity
Hydroxycobalamin	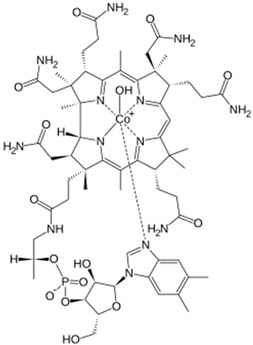	Approved	5 g administered by intravenous infusion over 15 min
Cobinamide	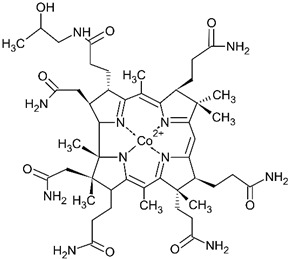	Experimental	Unknown
Dimethyl trisulfide (DMTS)	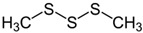	Experimental	Intramuscular injection (6.25–200 mg/kg) was given to rats 1 min after an oral dose of NaCN

## Data Availability

Not applicable.

## References

[1] Nath A.K., Shi X., Harrison D.L., Morningstar J.E., Mahon S., Chan A., Sips P., Lee J., MacRae C.A., Boss G.R. (2017). Cisplatin Analogs Confer Protection against Cyanide Poisoning. Cell Chem. Biol..

[2] Lawson-Smith P., Jansen E.C., Hilsted L., Hyldegaard O. (2010). Effect of Hyperbaric Oxygen Therapy on Whole Blood Cyanide Concentrations in Carbon Monoxide Intoxicated Patients from Fire Accidents. Scand. J. Trauma Resusc. Emerg. Med..

[3] Anseeuw K., Delvau N., Burillo-Putze G., De Iaco F., Geldner G., Holmström P., Lambert Y., Sabbe M. (2013). Cyanide Poisoning by Fire Smoke Inhalation: A European Expert Consensus. Eur. J. Emerg. Med..

[4] Hamel J. (2011). A Review of Acute Cyanide Poisoning with a Treatment Update. Crit Care Nurse.

[5] Lawson-Smith P., Jansen E.C., Hyldegaard O. (2011). Cyanide Intoxication as Part of Smoke Inhalation-a Review on Diagnosis and Treatment from the Emergency Perspective. Scand. J. Trauma Resusc. Emerg. Med..

[6] Morris A.A., Page R.L., Baumgartner L.J., Mueller S.W., MacLaren R., Fish D.N., Kiser T.H. (2017). Thiocyanate Accumulation in Critically Ill Patients Receiving Nitroprusside Infusions. J. Intensive Care Med..

[7] Jaszczak E., Polkowska Ż., Narkowicz S., Namieśnik J. (2017). Cyanides in the Environment—Analysis—Problems and Challenges. Environ. Sci. Pollut. Res..

[8] Liang C.J., Li J.Y., Ma C.J. (2014). Review on Cyanogenic Bacteria for Gold Recovery from E-Waste. Adv. Mater. Res..

[9] Økland O.P., Nakstad E.R., Opdahl H. (2020). Carbon Monoxide and Cyanide Gas Poisoning in Fires. Tidsskr. Nor. Laegeforen..

[10] Baud F.J. (2007). Cyanide: Critical Issues in Diagnosis and Treatment. Hum. Exp. Toxicol..

[11] Borron S.W. (2006). Recognition and Treatment of Acute Cyanide Poisoning. J. Emerg. Nurs..

[12] EFSA Panel on Contaminants in the Food Chain (CONTAM) (2016). Acute Health Risks Related to the Presence of Cyanogenic Glycosides in Raw Apricot Kernels and Products Derived from Raw Apricot Kernels. EFSA J..

[13] Newhouse K., Chiu N. (2010). Toxicological Review of Hydrogen Cyanide and Cyanide Salts.

[14] Rockwood G.A., Platoff Jr G.E., Salem H. (2019). Cyanides: Toxicology, Clinical Presentation, and Medical Management. Chemical Warfare Agents: Biomedical and Psychological Effects, Medical Countermeasures, and Emergency Response.

[15] Lorke D. (1983). A New Approach to Practical Acute Toxicity Testing. Arch. Toxicol..

[16] Sterner R.T. (1979). Effects of Sodium Cyanide and Diphacinone in Coyotes (*Canis latrans*): Applications as Predacides in Livestock Toxic Collars. Bull. Environ. Contam. Toxicol..

[17] Smyth Jr H.F., Carpenter C.P., Weil C.S., Pozzani U.C., Striegel J.A., Nycum J.S. (1969). Range-Finding Toxicity Data: List VII. Am. Ind. Hyg. Assoc. J..

[18] Graham J., Traylor J. (2022). Cyanide Toxicity. StatPearls [Internet].

[19] Lachowicz J.I., Milia S., Jaremko M., Oddone E., Cannizzaro E., Cirrincione L., Malta G., Campagna M., Lecca L.I. (2022). Cooking Particulate Matter: A Systematic Review on Nanoparticle Exposure in the Indoor Cooking Environment. Atmosphere.

[20] Poulton J.E. (1990). Cyanogenesis in Plants. Plant Physiol..

[21] Tylleskär T., Banea M., Bikangi N., Fresco L., Persson L.A., Rosling H. (1991). Epidemiological Evidence from Zaire for a Dietary Etiology of Konzo, an Upper Motor Neuron Disease. Bull. World Health Organ..

[22] Barrett M.D.P., Alexander J.C., Hill D.C. (1978). Effect of Linamarin on Thiocyanate Production and Thyroid Activity in Rats. J. Toxicol. Environ. Health Part A Curr. Issues.

[23] Buonvino S., Arciero I., Melino S. (2022). Thiosulfate-Cyanide Sulfurtransferase a Mitochondrial Essential Enzyme: From Cell Metabolism to the Biotechnological Applications. Int. J. Mol. Sci..

[24] Cressey P., Reeve J. (2019). Metabolism of Cyanogenic Glycosides: A Review. Food Chem. Toxicol..

[25] Hidayati N., Juhaeti T., Syarif F. (2009). Mercury and Cyanide Contaminations in Gold Mine Environment and Possible Solution of Cleaning up by Using Phytoextraction. Hayati J. Biosci..

[26] Chen M.-H., Yu X.-Z., Feng Y.-X. (2021). Tracing the Pollution and Human Risks of Potentially Toxic Elements in Agricultural Area Nearby the Cyanide Baths from an Active Private Gold Mine in Hainan Province, China. Environ. Geochem. Health.

[27] Kuliahsari D.E., Sari I.N.I., Estiasih T. (2021). Cyanide Detoxification Methods in Food: A Review. Proc. IOP Conf. Ser. Earth Environ..

[28] Alvillo-Rivera A., Garrido-Hoyos S., Buitron G., Thangarasu-Sarasvathi P., Rosano-Ortega G. (2021). Biological Treatment for the Degradation of Cyanide: A Review. J. Mater. Res. Technol..

[29] Leanderson P. (2019). Isocyanates and Hydrogen Cyanide in Fumes from Heated Proteins and Protein-rich Foods. Indoor Air.

[30] Wang Z., Nicholls S.J., Rodriguez E.R., Kummu O., Hörkkö S., Barnard J., Reynolds W.F., Topol E.J., DiDonato J.A., Hazen S.L. (2007). Protein Carbamylation Links Inflammation, Smoking, Uremia and Atherogenesis. Nat. Med..

[31] Benson S.M., Maskrey J.R., Nembhard M.D., Unice K.M., Shirley M.A., Panko J.M. (2019). Evaluation of Personal Exposure to Surgical Smoke Generated from Electrocautery Instruments: A Pilot Study. Ann. Work Expo. Health.

[32] Wachełko O., Chłopaś-Konowałek A., Zawadzki M., Szpot P. (2021). Old Poison, New Problem: Cyanide Fatal Intoxications Associated with Internet Shopping. J. Anal. Toxicol..

[33] Nishio T., Toukairin Y., Hoshi T., Arai T., Nogami M. (2021). A Fatal Poisoning Case of Acetone Cyanohydrin and Citalopram. Leg. Med..

[34] Leavesley H.B., Li L., Prabhakaran K., Borowitz J.L., Isom G.E. (2008). Interaction of Cyanide and Nitric Oxide with Cytochrome c Oxidase: Implications for Acute Cyanide Toxicity. Toxicol. Sci..

[35] Stotz E., Altschul A.M., Hogness T.R. (1938). The Cytochrome C-Cytochrome Oxidase Complex. J. Biol. Chem..

[36] Yoshikawa S., Muramoto K., Shinzawa-Itoh K. (2011). The O_2_ Reduction and Proton Pumping Gate Mechanism of Bovine Heart Cytochrome *c* Oxidase. Biochim. Biophys. Acta (BBA)-Bioenerg..

[37] Jones M.G., Bickar D., Wilson M.T., Brunori M., Colosimo A., Sarti P. (1984). A Re-Examination of the Reactions of Cyanide with Cytochrome c Oxidase. Biochem. J..

[38] Pearson R.G. (1995). The HSAB Principle—More Quantitative Aspects. Inorganica Chim. Acta.

[39] Aaseth J., Crisponi G., Anderson O. (2016). Chelation Therapy in the Treatment of Metal Intoxication.

[40] Gibson Q.H., Greenwood C. (1963). Reactions of Cytochrome Oxidase with Oxygen and Carbon Monoxide. Biochem. J..

[41] Nicholls P. (1975). The Effect of Sulphide on Cytochrome Aa3. Isosteric and Allosteric Shifts of the Reduced Alpha-Peak. Biochim. Biophys. Acta.

[42] Pearce L.L., Bominaar E.L., Hill B.C., Peterson J. (2003). Reversal of Cyanide Inhibition of Cytochrome c Oxidase by the Auxiliary Substrate Nitric Oxide: An Endogenous Antidote to Cyanide Poisoning?. J. Biol. Chem..

[43] Hendry-Hofer T.B., Ng P.C., Witeof A.E., Mahon S.B., Brenner M., Boss G.R., Bebarta V.S. (2019). A Review on Ingested Cyanide: Risks, Clinical Presentation, Diagnostics, and Treatment Challenges. J. Med. Toxicol..

[44] Mitchella B.L., Bhandaria R.K., Bebartab V.S., Rockwoodc G.A., Bossd G.R., Loguea B.A. (2013). Toxicokinetic Profiles Of-Ketoglutarate Cyanohydrin, a Cyanide Detoxification Product, Following Exposure to Potassium Cyanide. Toxicol. Lett..

[45] Sousa A.B., Manzano H., Soto-Blanco B., Górniak S.L. (2003). Toxicokinetics of Cyanide in Rats, Pigs and Goats after Oral Dosing with Potassium Cyanide. Arch. Toxicol..

[46] de Sousa A.B., Górniak S.L. (2014). Toxicokinetic Aspects of Thiocyanate after Oral Exposure to Cyanide in Female Wistar Rats in Different Physiological States. Drug Chem. Toxicol..

[47] Sabourin P.J., Kobs C.L., Gibbs S.T., Hong P., Matthews C.M., Patton K.M., Sabourin C.L., Wakayama E.J. (2016). Characterization of a Mouse Model of Oral Potassium Cyanide Intoxication. Int. J. Toxicol..

[48] Bhandari R.K., Oda R.P., Petrikovics I., Thompson D.E., Brenner M., Mahon S.B., Bebarta V.S., Rockwood G.A., Logue B.A. (2014). Cyanide Toxicokinetics: The Behavior of Cyanide, Thiocyanate and 2-Amino-2-Thiazoline-4-Carboxylic Acid in Multiple Animal Models. J. Anal. Toxicol..

[49] Leuschner J., Winkler A., Leuschner F. (1991). Toxicokinetic Aspects of Chronic Cyanide Exposure in the Rat. Toxicol. Lett..

[50] Dirikolu L., Hughes C., Harkins D., Boyles J., Bosken J., Lehner F., Troppmann A., McDowell K., Tobin T., Sebastian M.M. (2003). The Toxicokinetics of Cyanide and Mandelonitrile in the Horse and Their Relevance to the Mare Reproductive Loss Syndrome. Toxicol. Mech. Methods.

[51] Djerad A., Monier C., Houzé P., Borron S.W., Lefauconnier J.-M., Baud F.J. (2001). Effects of Respiratory Acidosis and Alkalosis on the Distribution of Cyanide into the Rat Brain. Toxicol. Sci..

[52] Tshala-Katumbay D., Mumba N., Okitundu L., Kazadi K., Banea M., Tylleskär T., Boivin M., Muyembe-Tamfum J.J. (2013). Cassava Food Toxins, Konzo Disease, and Neurodegeneration in Sub-Sahara Africans. Neurology.

[53] Willemin M.-E., Lumen A. (2017). Thiocyanate: A Review and Evaluation of the Kinetics and the Modes of Action for Thyroid Hormone Perturbations. Crit. Rev. Toxicol..

[54] Vogel S.N., Sultan T.R., Ten Eyck R.P. (1981). Cyanide Poisoning. Clin. Toxicol..

[55] Lee J., Mahon S.B., Mukai D., Burney T., Katebian B.S., Chan A., Bebarta V.S., Yoon D., Boss G.R., Brenner M. (2016). The Vitamin B 12 Analog Cobinamide Is an Effective Antidote for Oral Cyanide Poisoning. J. Med. Toxicol..

[56] Konstantatos A., Kumar M.S., Burrell A., Smith J. (2017). An Unusual Presentation of Chronic Cyanide Toxicity from Self-Prescribed Apricot Kernel Extract. Case Rep..

[57] Llorens J., Soler-Martín C., Saldaña-Ruíz S., Cutillas B., Ambrosio S., Boadas-Vaello P. (2011). A New Unifying Hypothesis for Lathyrism, Konzo and Tropical Ataxic Neuropathy: Nitriles Are the Causative Agents. Food Chem. Toxicol..

[58] Cheok S.S. (1978). Acute Cassava Poisoning in Children in Sarawak. Trop. Dr..

[59] Tylleskär T., Rosling H., Banea M., Bikangi N., Cooke R.D., Poulter N.H. (1992). Cassava Cyanogens and Konzo, an Upper Motoneuron Disease Found in Africa. Lancet.

[60] Oyewopo A., Adeleke O., Johnson O., Akingbade A. (2021). Quercetin Upregulates CREM Gene Expression in Cyanide-Induced Endocrine Dysfunction. Heliyon.

[61] d’Anglemont de Tassigny X., Colledge W.H. (2010). The Role of Kisspeptin Signaling in Reproduction. Physiology.

[62] Shiva M., Gautam A.K., Verma Y., Shivgotra V., Doshi H., Kumar S. (2011). Association between Sperm Quality, Oxidative Stress, and Seminal Antioxidant Activity. Clin. Biochem..

[63] Zacarias C.H., Esteban C., Rodrigues G.L., Nascimento E.d.S. (2017). Occupational exposure to hydrogen cyanide during large-scale cassava processing, in Alagoas State, Brazil. Cad. De Saúde Pública.

[64] Ferrari L.A., Giannuzzi L. (2015). Assessment of Carboxyhemoglobin, Hydrogen Cyanide and Methemoglobin in Fire Victims: A Novel Approach. Forensic Sci. Int..

[65] Lappas N.T., Lappas C.M. (2021). Forensic Toxicology: Principles and Concepts.

[66] Odoul M., Fouillet B., Nouri B., Chambon R., Chambon P. (1994). Specific Determination of Cyanide in Blood by Headspace Gas Chromatography. J. Anal. Toxicol..

[67] Lindsay A.E., Greenbaum A.R., O’Hare D. (2004). Analytical Techniques for Cyanide in Blood and Published Blood Cyanide Concentrations from Healthy Subjects and Fire Victims. Anal. Chim. Acta.

[68] Nagy A., Nagy G. (1993). Amperometric Air Gap Cell for the Measurement of Free Cyanide. Anal. Chim. Acta.

[69] Pihlar B., Kosta L. (1980). Determination of Cyanides by Continuous Distillation and Flow Analysis with Cylindrical Amperometric Electrodes. Anal. Chim. Acta.

[70] Kim M., Jee S.-C., Kim S., Hwang K.-H., Sung J.-S. (2021). Identification and Characterization of MRNA Biomarkers for Sodium Cyanide Exposure. Toxics.

[71] Stamyr K., Mörk A.K., Johanson G. (2015). Physiologically Based Pharmacokinetic Modeling of Hydrogen Cyanide Levels in Human Breath. Arch. Toxicol..

[72] Rodgers Jr G.C., Condurache C.T. (2010). Antidotes and Treatments for Chemical Warfare/Terrorism Agents: An Evidence-Based Review. Clin. Pharmacol. Ther..

[73] Henretig F.M., Kirk M.A., McKay C.A. (2019). Hazardous Chemical Emergencies and Poisonings. N. Engl. J. Med..

[74] Leavesley H.B., Li L., Mukhopadhyay S., Borowitz J.L., Isom G.E. (2010). Nitrite-Mediated Antagonism of Cyanide Inhibition of Cytochrome c Oxidase in Dopamine Neurons. Toxicol. Sci..

[75] Haouzi P., Gueguinou M., Sonobe T., Judenherc-Haouzi A., Tubbs N., Trebak M., Cheung J., Bouillaud F. (2018). Revisiting the Physiological Effects of Methylene Blue as a Treatment of Cyanide Intoxication. Clin. Toxicol..

[76] Marrs T.C., Thompson J.P. (2016). The Efficacy and Adverse Effects of Dicobalt Edetate in Cyanide Poisoning. Clin. Toxicol..

[77] Nagler J., Provoost R.A., Parizel G. (1978). Hydrogen Cyanide Poisoning: Treatment with Cobalt EDTA. J. Occup. Med..

[78] Kiernan E., Carpenter J.E., Dunkley C.A., Koch D., Morgan B.W., Steck A.R., Murray B.P. (2020). Elevated Methemoglobin Levels in a Patient Treated with Hydroxocobalamin After Suspected Cyanide Exposure. J. Emerg. Med..

[79] Jiwani A.Z., Bebarta V.S., Cancio L.C. (2018). Acquired Methemoglobinemia after Hydroxocobalamin Administration in a Patient with Burns and Inhalation Injury. Clin. Toxicol..

[80] Dang T., Nguyen C., Tran P.N. (2017). Physician Beware: Severe Cyanide Toxicity from Amygdalin Tablets Ingestion. Case Rep. Emerg. Med..

[81] Meredith T.J., Jacobsen D., Haines J.A., Berger J.C., van Heijst A.N.P. (2009). IPCS/CEC Evaluation of Antidotes Series. Anal. Methods.

[82] Suman S.G., Gretarsdottir J.M. (2019). Chemical and Clinical Aspects of Metal-Containing Antidotes for Poisoning by Cyanide. Met. Ions Life Sci..

[83] Petrikovics I., Budai M., Kovacs K., Thompson D.E. (2015). Past, Present and Future of Cyanide Antagonism Research: From the Early Remedies to the Current Therapies. World J. Methodol..

[84] Chan A., Lee J., Bhadra S., Bortey-Sam N., Hendry-Hofer T.B., Bebarta V.S., Mahon S.B., Brenner M., Logue B., Pilz R.B. (2022). Development of Sodium Tetrathionate as a Cyanide and Methanethiol Antidote. Clin. Toxicol..

[85] Ng P.C., Hendry-Hofer T.B., Witeof A.E., Mahon S.B., Brenner M., Boss G.R., Bebarta V.S. (2019). Efficacy of Oral Administration of Sodium Thiosulfate and Glycine in a Large, Swine Model of Oral Cyanide Toxicity. Ann. Emerg. Med..

[86] Rice N.C., Rauscher N.A., Wilkins W.L., Lippner D.S., Rockwood G.A., Myers T.M. (2019). Behavioural and Physiological Assessments of Dimethyl Trisulfide Treatment for Acute Oral Sodium Cyanide Poisoning. Basic Clin. Pharmacol. Toxicol..

[87] Medici S., Peana M., Nurchi V.M., Lachowicz J.I., Crisponi G., Zoroddu M.A. (2014). Noble Metals in Medicine: Latest Advances. Coord. Chem. Rev..

[88] Harrington J.M., Jones S.B., Hancock R.D. (2005). Determination of Formation Constants for Complexes of Very High Stability: Log Β4 for the [Pd (CN) 4] 2−Ion. Inorganica Chim. Acta.

[89] Behymer M.M., Mo H., Fujii N., Suresh V., Chan A., Lee J., Nath A.K., Saha K., Mahon S.B., Brenner M. (2022). Identification of Platinum (II) Sulfide Complexes Suitable as Intramuscular Cyanide Countermeasures. Chem. Res. Toxicol..

